# The ‘Sandalwood Leopard’: A Rare Colour Morph of the Indian Leopard ( *Panthera pardus fusca* ) From Southern India

**DOI:** 10.1002/ece3.74687

**Published:** 2026-11-13

**Authors:** Sanjay Gubbi, Shravan Suthar, H. C. Poornesha, Sandesh Appu Naik

**Affiliations:** ^1^ Holématthi Nature Foundation Bengaluru India

**Keywords:** colour morph, erythrism, Indian leopard, Karnataka, *Panthera pardus*, phenotype

## Abstract

We report a female Indian leopard ( *Panthera pardus fusca* ) exhibiting a rare ‘Sandalwood’ colour morph from a camera‐trap survey conducted in southern India between December 2025 and January 2026. The study area comprised dry deciduous and scrub habitats. Among five individuals identified in the study area, this individual was recorded on nine occasions across six camera‐trap locations. Across a long‐term dataset comprising 926 individually identified leopards from 148,853 camera‐trap days collected over 14 years of surveys across Karnataka, this represents the only scientific record of this phenotype. The individual displayed a distinctive combination of traits, including lighter pelage, pale brown rosettes, and reduced pigmentation in exposed skin. Although these features are clearly distinguishable from the typical phenotype, the underlying genetic basis remains unknown and would require molecular analysis from non‐invasive samples. In the absence of such evidence, we conservatively classify this individual as a rare colour morph. As the first scientifically documented record of this phenotype from Karnataka, we propose the term ‘Sandalwood leopard’ as a regionally contextualised descriptor reflecting the state's natural and cultural heritage.

## Graphical abstract

We report a rare ‘sandalwood’ colour morph in a female Indian leopard from southern Karnataka, recorded nine times during camera‐trap surveys and uniquely identified within a 14‐year dataset of 926 individuals. Characterised by lighter pelage and reduced pigmentation, this phenotype is provisionally classified as a rare colour morph, and we propose the term ‘Sandalwood leopard’ as a regionally contextualised descriptor.

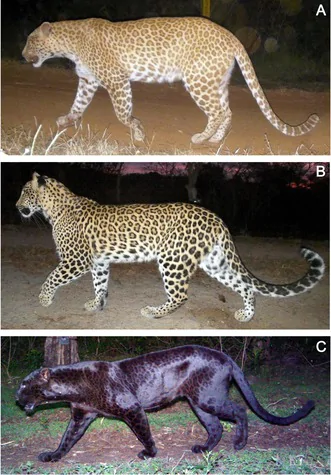

## 1 Introduction

Coat colour dilution in big cats has been attributed to genetic variants in multiple genes (Tensen et al. 2022). More broadly, coat colouring variations arise from gene mutations as well as environmental and organismal factors (Jain and Verma 2024).

Leopards ( *Panthera pardus* ), a wide ranging large spotted felid assessed as Vulnerable by the IUCN (Stein et al. 2025), exhibit genetically determined colour variation. In addition to the ‘normal’ or ‘wild‐type’ pheno‐type, melanism is a commonly observed colour variant in this species (Figure 1). More recently, a rare colour morph characterised by pale red‐brown coat, rosettes that are red or pale brown rather than of black, a pink nose, blue eyes and pink paw pads has been documented. This colour morph has been referred to as ‘strawberry leopard’, or ‘red leopard’ (Searle et al. 2024). Such colour morph (erythristic) individuals are exceptionally rare in the wild and have been reported only a handful of times in African leopards (*P.p.pardus*) from South Africa (Dell'Amore 2012; Pirie et al. 2016), Tanzania (Searle et al. 2024), and Indian leopards ( *Panthera pardus fusca* Divyabhanusinh 1993). This leopard phenotype, first described from South Africa as erythristic (Pirie et al. 2016), has more recently been suggested to be linked to albinism‐related pathways (Tensen et al. 2022).

**FIGURE 1:**
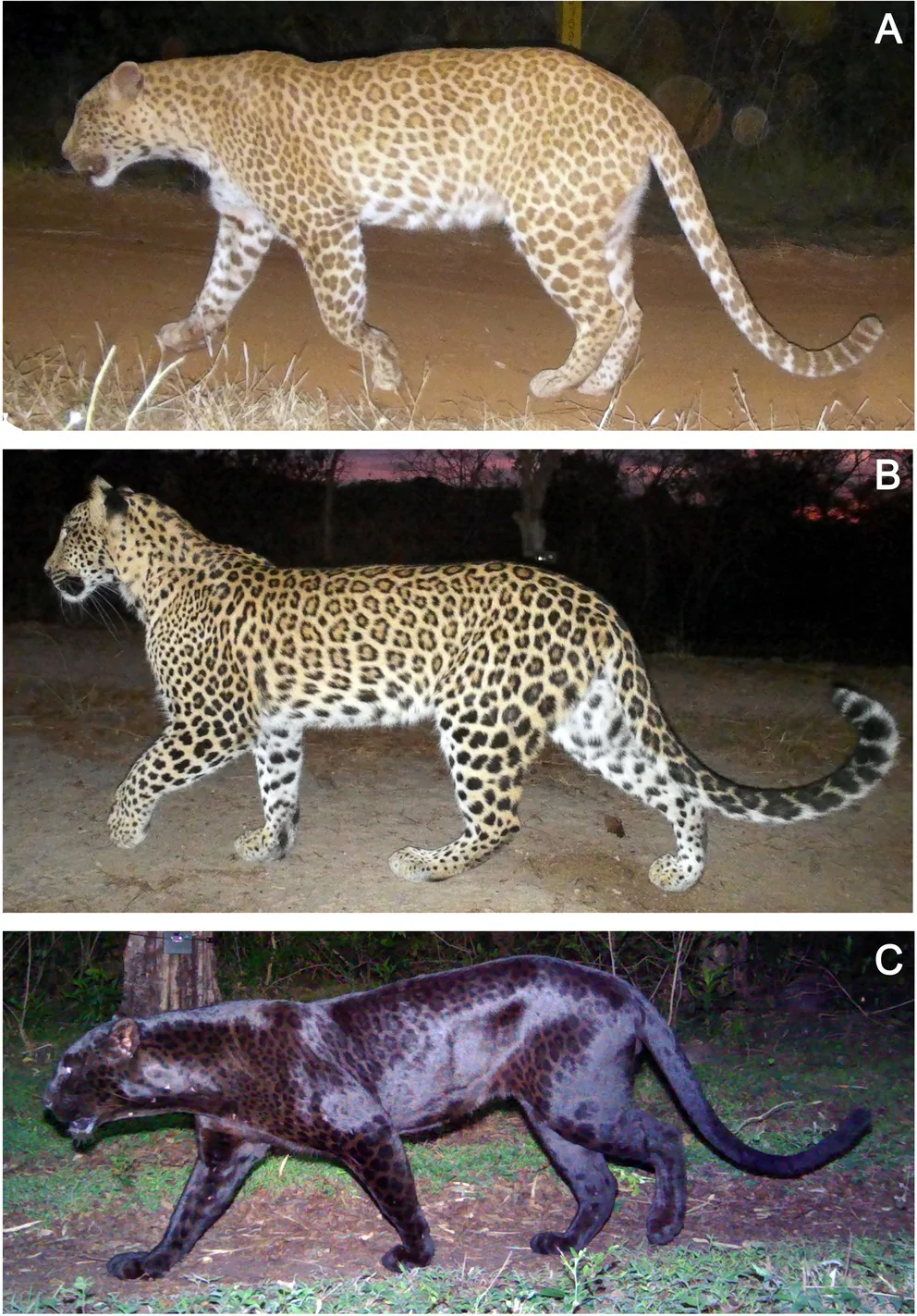
Photo of the ʻSandalwood leopard’ in Sogi Reserved Forest (a) in comparison to a normal phenotype leopard (b) and melanistic leopard (c).

India hosts a large population of the Indian leopard and is therefore a key country for the conservation of this subspecies, contributing significantly to the conservation of the species as a whole. Melanism is widespread within this subspecies in India and has been well documented (da Silva et al. 2017; Mahabal et al. 2019). Although anecdotal accounts of white or light‐coloured leopards have been documented in both scientific and popular literature for over a century (Divyabhanusinh 1993), there has been only one confirmed sighting, supported by photographic evidence, of the pale red‐brown colour morph in Rajasthan, India (Dsouza 2021). To date, this colour morph has not been recorded from any other country in Asia. The geographic distribution of this variant remains poorly documented; therefore, reporting such occurrences is important for mapping its distribution and improving our broader understanding of leopard conservation.

## 2 Materials and Methods

This study was conducted in Sogi Reserved Forest (33.2 km^2^, 14°52′20.62″ N—14°54′26.81″ N, 75°57′42.61″ E—76°0′59.95″ E, Figure 2) located in the state of Karnataka (191,791km^2^), southern India. Sogi Reserved Forest is characterised as a dry deciduous scrub ecosystem. The region experiences a semi‐arid climate, with a mean annual rainfall of 700 mm and temperatures ranging from 18°C to 40°C.

**FIGURE 2:**
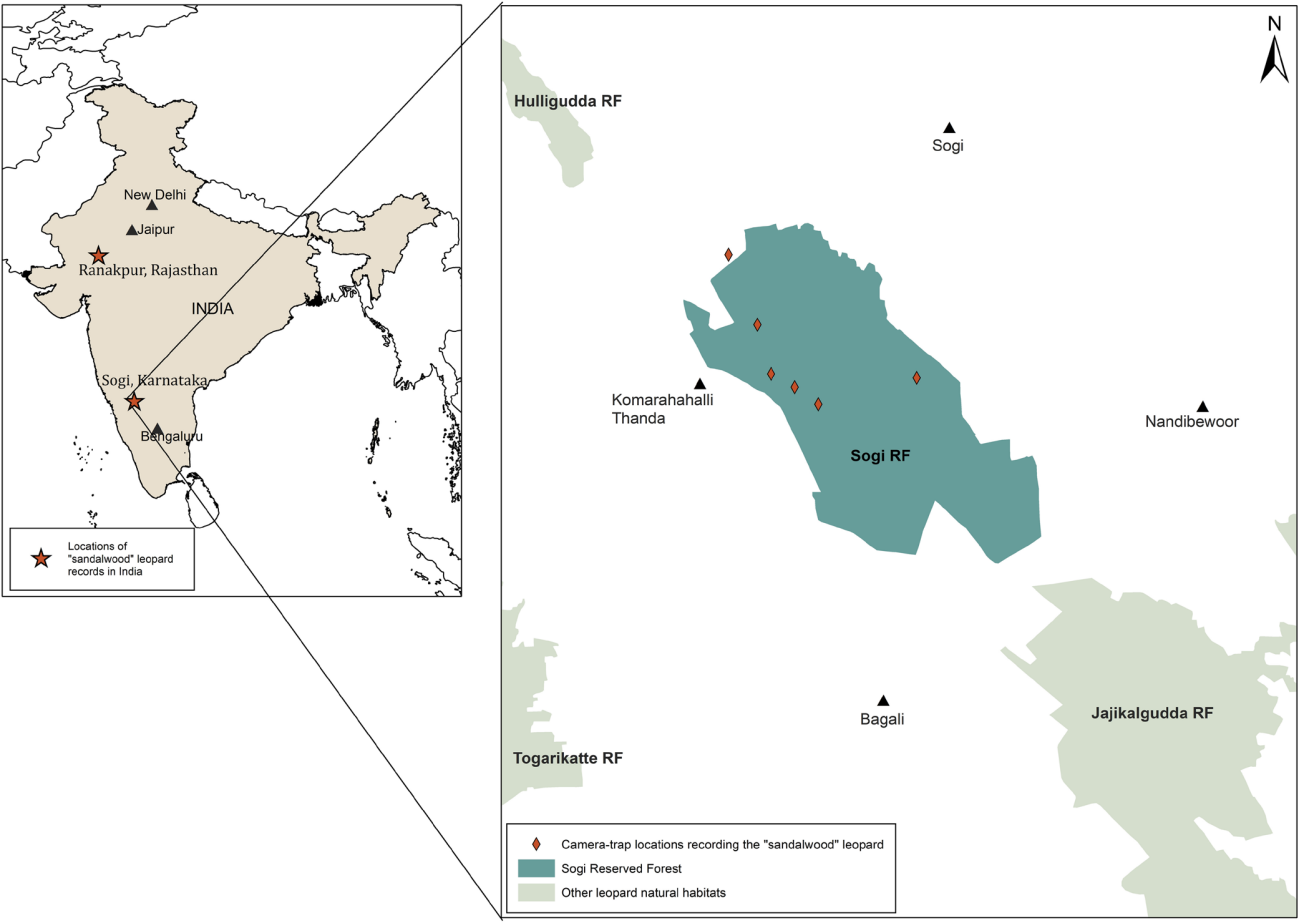
Map of the study area showing the locations of the ʻSandalwood leopard’ captures in Sogi Reserved Forest, southern India, and the previous documentation in India.

Between December 2025 and January 2026, we conducted camera trap surveys to collect data for estimating leopard abundance and density using spatially explicit capture‐recapture (SECR) methodology. Motion‐activated camera traps equipped with xenon white flash (Spartan Lumen SR3‐CX, Panthera‐V4 and V6 models) were deployed across the study area. Each location consisted of two camera traps on each side of the forest roads.

A total of 34 paired camera trap stations were established, primarily along forest roads. Each station consisted of two cameras positioned on opposite sides of the road to capture bilateral images of passing individual leopards. Camera stations were active for 16 consecutive days. We adopted a 16‐day sampling period to minimise potential violations of demographic closure through births and deaths, consistent with its suitability for leopard density estimation in our previous surveys.

Individual leopards were identified from camera trap photographs based on their unique rosette patterns, which allow reliable differentiation of individuals for capture–recapture analyses.

## 3 Results

Across the study site, we deployed 34 paired camera traps over 16 days, resulting in a total sampling effort of 544 camera‐trap days. These cameras yielded 138 images of leopards, from which five individuals were identified (two females, one male, and two cubs). The SECR analysis estimated a density of 8.33 leopards/100 km^2^ (SE ±3.27) in the Sogi cluster. Among these, we documented one female leopard exhibiting unusual coloration.

Comparison of these images with those of normally pigmented leopards, based on both diurnal and nocturnal photographs, revealed a lighter pelage characterised by pale brown rosettes (Figure 1), a pink nasal planum, light brown pigmentation on the posterior surface of the pinnae, and pink pigmentation on the ventral surface of the paws (Figure 3). However, the presence of blue irises could not be confirmed. No image processing was applied to normalise colours between images of the ‘Sandalwood’ individual and normally phenotype leopards (Figure 1). This individual was recorded on nine occasions across six different camera‐trap locations (see Table 1 and Figure 3 for details). Of these nine capture events, the animal was photographed alone in six instances and accompanied by two normal phenotype cubs in three instances. The cubs appeared to be about 10–12 months and were observed following their mother.

**FIGURE 3:**
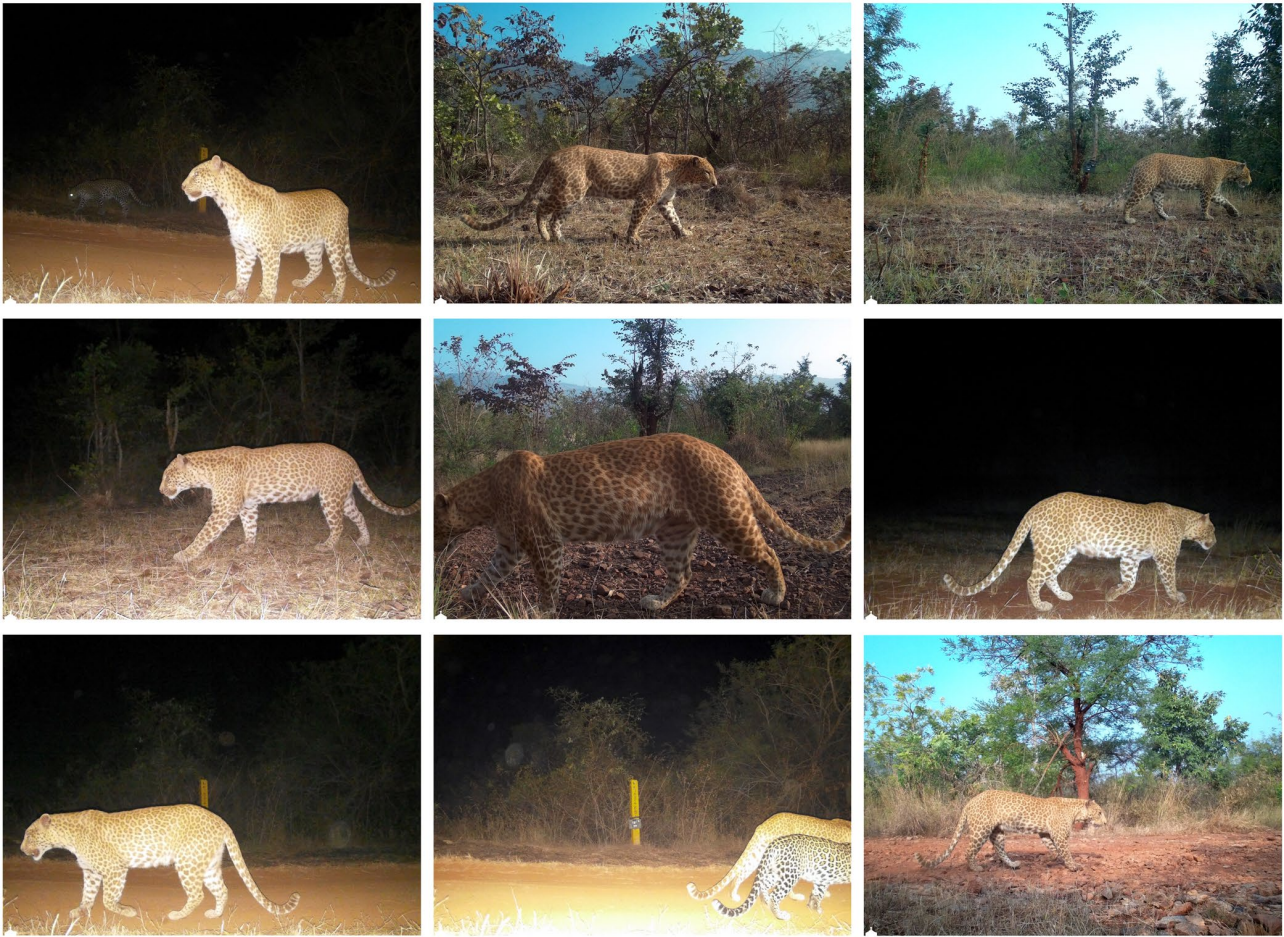
All photographs of the 'Sandalwood leopard' from nine capture events in Sogi Reserved Forest, southern India.

**TABLE 1:** Camera‐trap capture events of the female ‘Sandalwood leopard’ ( *Panthera pardus fusca* ) in Sogi Reserved Forest, Karnataka, southern India. Asterisks indicate events in which the female was photographed with cubs.

Capture event	Date	Time (h)	Lat, long	Elevation (m)	No. of images
1*	26th December 2025	22:20	14.9395, 75.9486	615.637	17
2	27th December 2025	09:26	14.9243, 75.9549	613.389	4
3	27th December 2025	17:19	14.9136, 75.9579	591.146	2
4*	28th December 2025	03:48	14.9243, 75.9549	613.389	4
5	29th December 2025	08:08	14.9107, 75.9630	597.936	3
6	29th December 2025	03:56	14.9070, 75.9682	609.649	1
7	30th December 2025	19:55	14.9395, 75.9486	615.637	1
8*	30th December 2025	21:11	14.9395, 75.9486	615.637	2
9	31st December 2025	09:07	14.9127, 75.9896	697.921	3

* Camera‐trapped with cubs.

From our cumulative dataset comprising 926 individually identified leopards across 6,577 km^2^ and a total camera‐trap effort of 148,853 trap days, this represents the only record of this colour morph. During the study on leopards over the past 14 years, this is the first documented occurrence of this phenotype in Karnataka. Hence we propose the designation ‘Sandalwood leopard,’ assigning a regionally contextualised identity that reflects the state's natural and cultural heritage.

## 4 Discussion

To date, no cases of this erythristic colour morph have been reported in Asia outside India, making this the second documented record of a ‘Sandalwood leopard’ in Asia. This represents the second documented record across all leopard subspecies occurring in Asia, rather than solely within *P. p. fusca*. The current record is at a distance approximately 1,300 km from the closet existing confirmed record from the state of Rajasthan (Figure 2). Interestingly, both locations are semi‐arid, scrub habitats. This is also the first scientific record for the state of Karnataka which holds substantial populations of leopards. This colour morph, in addition to being ultra rare was also documented for this first time within our dataset.

The unusually pale, reddish appearance of this leopard suggests a rare coat‐colour variation that may be consistent with conditions such as hypomelanism or erythrism, both of which are naturally occurring genetic traits identified in wild mammals. At present, this assessment is based on photographic evidence and visual characteristics. While these features are distinctive, definitive confirmation of the exact genetic mechanism would require molecular analysis using DNA from non‐invasive samples such as scat or hair. Until such genetic evidence is available, it is scientifically appropriate to describe this animal as a rare colour morph, rather than assign a specific genetic condition. Importantly, this variation does not indicate poor health or abnormality, and the leopard appears to be a normal, free‐ranging individual. No abnormalities in behaviour or condition were observed.

No other scientific records of this phenotype are available on records between these two records.

The ‘Sandalwood leopard’ could have arisen in Sogi Reserved Forest as a result of heterozygote deficiency due to inbreeding (Tensen and Fischer 2024; Searle et al. 2024) as this leopard population is isolated.

Two patches of natural leopard habitat—Jajikalguddi (38.13 km^2^) and Jalkalgudda (3.52 km^2^)—lie near Sogi Reserved Forest, separated from it by just 850 m of agricultural fields. Together with Sogi Reserved Forest, they form a small habitat cluster (74.85 km^2^). The nearest patches of natural habitat outside the Sogi cluster are at least 6.9 km away, separated by agricultural fields and dense human settlements that may restrict leopard movement. This fragmentation could reduce functional connectivity, potentially limiting gene flow and increasing the risk of inbreeding among leopards within the cluster.

Although crops such as sugarcane and maize can provide cover for leopards to inhabit and move through agricultural landscapes (Gubbi et al. 2020), these crops are either absent from the intervening landscape or cultivated only seasonally. They therefore do not provide consistent, year‐round cover for movement between distant habitat patches.

Such inbreeding effects are seen in king cheetah (*Acinonyx jubatus*), white lion ( *Panthera leo* ) and jaguars ( *Panthera onca* ) (Haag et al. 2010; Sunquist and Sunquist 2014; Pirie et al. 2016) colour morphs.

In leopards, young males do not need to disperse far from their mothers, which increases chances of inbreeding and thus the chance of rare recessive alleles being expressed (Pirie et al. 2016; Tensen et al. 2022). This calls for attention about larger contiguous landscapes for leopard conservation in India, which has a high leopard population.

Future studies could use DNA from non‐invasively collected scats or shed hair to investigate pigmentation‐related candidate genes, including MC1R, ASIP, and TYRP1. TYRP1 warrants particular attention because a mutation in this gene has been linked to the red colour morph in South African leopards (Tensen et al. 2022). Comparisons with normally coloured individuals could help identify variants associated with the ‘Sandalwood’ phenotype, although further validation would be required to establish causality.

This was the first scientific record of ‘Sandalwood leopard’ from our studies across the state of Karnataka covering 148,853 camera trapping efforts. Based on this extensive work we conclude that this colour morph is extremely rare in our state as we feel there is unlikely to be a reporting bias.

## Author Contributions

**Sanjay Gubbi:** conceptualization (lead), data curation (supporting), formal analysis (lead), funding acquisition (lead), investigation (equal), methodology (lead), project administration (equal), resources (lead), software (supporting), supervision (lead), validation (equal), visualization (supporting), writing – original draft (lead), writing – review and editing (lead). **Shravan Suthar:** conceptualization (supporting), data curation (equal), formal analysis (equal), funding acquisition (supporting), investigation (equal), methodology (supporting), project administration (equal), resources (supporting), software (equal), supervision (equal), validation (equal), visualization (supporting), writing – original draft (supporting), writing – review and editing (supporting). **H. C. Poornesha:** data curation (equal), project administration (equal), visualization (equal). **Sandesh Appu Naik:** investigation (equal), methodology (equal), project administration (equal), supervision (equal).

## Funding

The study was funded by The Habitats Trust. Additional support was provided by the British Asian Trust.

## Ethics Statement

The study was carried out with permissions from the Karnataka Forest Department.

## Conflicts of Interest

The authors declare no conflicts of interest.

## Data Availability

All capture data and images of the ‘sandalwood leopard’ can be found in Table 1 and Figures 1, 2, 3. We can share any other field data to confirm this event.

## References

[ece374687-bib-0001] da Silva, L. G. , K. Kawanishi , P. Henschel , et al. 2017. “Mapping Black Panthers: Macroecological Modeling of Melanism in Leopards ( *Panthera pardus* ).” PLoS One 12, no. 4: e0170378. 10.1371/journal.pone.0170378. PMC5381760 28379961

[ece374687-bib-0002] Dell'Amore, C. 2012. “Strawberry Leopard Discovered–A First.” Viewed 01 December 2015. http://news.nationalgeographic.com/news/2012/04/120412‐strawberry‐leopard‐south‐africaanimals‐science/.

[ece374687-bib-0003] Divyabhanusinh, C. 1993. “On Mutant Leopards *Panthera pardus* From India.” Journal of the Bombay Natural History Society 90: 88–89.

[ece374687-bib-0004] Dsouza, C. 2021. “In a First, Rare Pink Leopard Sighted in Rajasthan.” The Times of India. https://timesofindia.indiatimes.com/city/udaipur/in‐a‐1st‐in‐india‐leopard‐with‐strawberry‐coat‐seen‐in‐rajasthan/articleshow/87616657.cms.

[ece374687-bib-0005] Gubbi, S. , K. Sharma , and V. Kumara . 2020. “Every Hill Has Its Leopard: Patterns of Space Use by Leopards ( *Panthera pardus* ) in a Mixed Use Landscape in India.” PeerJ 8: 1–20. 10.7717/peerj.10072. PMC7548080 33083134

[ece374687-bib-0006] Haag, T. , A. S. Santos , D. A. Sana , et al. 2010. “The Effect of Habitat Fragmentation on the Genetic Structure of a Top Predator: Loss of Diversity and High Differentiation Among Remnant Populations of Atlantic Forest Jaguars ( *Panthera onca* ).” Molecular Ecology 19, no. 22: 4906–4921. 10.1111/j.1365-294X.2010.04856.x. 21040050

[ece374687-bib-0007] Jain, S. , and S. R. Verma . 2024. “Genetic Basis of Coat Colour Variation in Members of Genus Panthera.” Ecological Genetics and Genomics 30: 100300. 10.1016/j.egg.2024.100300.

[ece374687-bib-0008] Mahabal, A. , R. M. Sharma , R. N. Patil , and S. Jadhav . 2019. “Colour Aberration in Indian Mammals: A Review From 1886 to 2017.” Journal of Threatened Taxa 11, no. 6: 13690–13719. 10.11609/jott.3843.11.6.13690-13719.

[ece374687-bib-0009] Pirie, T. J. , R. L. Thomas , and M. D. Fellowes . 2016. “Erythristic Leopards *Panthera pardus* in South Africa.” Bothalia–African Biodiversity & Conservation 46, no. 1: 1–5. https://hdl.handle.net/10520/EJC195023.

[ece374687-bib-0010] Searle, C. E. , P. Strampelli , S. N. Parsais , et al. 2024. “New Record of Strawberry Leopard ( *Panthera pardus* ) in Selous Game Reserve, Tanzania.” Ecology and Evolution 14, no. 7: e11542. 10.1002/ece3.11542. PMC11229426 38979004

[ece374687-bib-0011] Stein, A. B. , P. Gerngross , H. Al Hikmani , et al. 2025. “ *Panthera pardus* (Amended Version of 2025 Assessment).” The IUCN Red List of Threatened Species 2025: e.T15954A286153337. 10.2305/IUCN.UK.2025-2.RLTS.T15954A286153337.en.

[ece374687-bib-0012] Sunquist, M. , and F. Sunquist . 2014. Wild Cats of the World. University of Chicago Press Ltd.

[ece374687-bib-0013] Tensen, L. , and K. Fischer . 2024. “Heterozygosity Is Low Where Rare Color Variants in Wild Carnivores Prevail.” Ecology and Evolution 14, no. 2: e10881. 10.1002/ece3.10881. PMC10847885 38327687

[ece374687-bib-0014] Tensen, L. , J. Power , G. Camacho , R. Godinho , B. Jansen van Vuuren , and K. Fischer . 2022. “Molecular Tracking and Prevalence of the Red Colour Morph Restricted to a Harvested Leopard Population in South Africa.” Evolutionary Applications 15, no. 6: 1028–1041. 10.1111/eva.13423. PMC9234631 35782007

